# The Expansion of the Economic Frontier and the Diffusion of Violence in the Amazon

**DOI:** 10.3390/ijerph120605862

**Published:** 2015-05-27

**Authors:** Patrícia Feitosa Souza, Diego Ricardo Xavier, Stephane Rican, Vanderlei Pascoal de Matos, Christovam Barcellos

**Affiliations:** 1 National School of Public Health (ENSP)-Fiocruz, Street Leopoldo Bulhões, 1480—Manguinhos, Rio de Janeiro 21041-210, Brazil; 2 Information Laboratory Health-LIS, Laboratory GIS, Avenue Brasil, 4365 Pavilhão Hai ty Moussatché, Sala 231-Manguinhos, Rio de Janeiro 21045-900, Brazil; E-Mails: diegoricardox@gmail.com (D.R.X.); vanderlei.matos@gmail.com (V.P.M.); srican@u-paris10.fr (S.R.); xris@fiocruz.br (C.B.); 3 Space and Territory Health Laboratory, Department of Geography, University Paris Ouest Nanterre Defense, 200 Avenue, Republic Nanterre, Paris 92000, France; E-Mail: stephane.rican@u-paris10.fr

**Keywords:** pioneer area, spatial diffusion of disease, violence, spatial analysis

## Abstract

Over the last few decades, the occupation of the Amazon and the expansion of large-scale economic activities have exerted a significant negative impact on the Amazonian environment and on the health of the Amazon’s inhabitants. These processes have altered the context of the manifestation of health problems in time and space and changed the characteristics of the spatial diffusion of health problems in the region. This study analyzed the relationships between the various economic processes of territorial occupation in the Amazon and the spatial diffusion of homicidal violence through the configuration of networks of production, as well as the movements of population and merchandise. Statistical data on violence, deforestation, the production of agricultural items, and socio-economic variables, georeferenced and available for the 771 municipalities of the Legal Amazon were used in this study. The results suggest that the diffusion of violence closely follows the economic expansion front, which is related to deforestation and livestock production but has little relation to grain production, demonstrating steps and typologies of recent occupation in the Amazon that promote violence. These spatial patterns reveal environmental and socio-economic macro-determinants that materialize in geographic space through the construction of highways and the formation of city networks.

## 1. Introduction

In the last few decades, an increasing number of homicides have been recorded in the Legal Amazon. Until the 1980s, the increase in homicides was centered in capital cities and major metropolitan areas, but after 2000, a diffusion of violence toward the peripheries and the interior of the states was observed. According to Waiselfisz [1], this change created two concurrent processes: the interiorization of homicides, which resulted in the diffusion of violence from large urban centers toward medium and small cities, and the migration of dynamic centers of violence from a limited number of metropolitan regions, which improved the efficiency of their safety apparatuses, to less-protected areas. The power and effective presence of the state in these areas was non-existent.

From a geographical perspective, an increase was observed in the number of violent deaths in smaller municipalities, particularly in regions where emergent forms of domination and control competed with the state for legitimacy in the use of violence to maintain power and authority over particular area [2]. In the Amazon, this conflictual framework largely occurs in disputes arising from political and economic actions and interests that hover around large agricultural enterprises in the consolidated pioneer area, the intermediary pioneer area (the deforestation arc), and the expanding pioneer area [3,4].

Conflicts over land possession, the predatory use of natural resources, slave labor, and urban segregation came to represent a distinctive characteristic of violence in the region in the mid-1990s.In this context, indigenous peoples, traditional populations, and small farmers have been the major victims of violence [5]. Such changes have a spatial logic because they occupy land and thus have different consequences on the spatial distribution of violence in the rural and urban Amazon, affecting certain areas and social groups at various intensities.

In the Brazilian Amazon, the phenomenon of homicidal violence arouses great concern because there is a high frequency of violent conflicts associated with land occupation where the agricultural frontier advances into the forest [6], affecting rural space and both large and small cities. The spatial diffusion of homicides is the result of a complex process that involves: (i) the spread of violence promoted by rivalries between rural workers and farming groups or firms disputing the ownership of land and natural resources; (ii) the concentration of vigilante groups and gunmen in small and medium cities in the region near the areas of agricultural expansion, ready to attack the political leadership of rural workers’ unions, and (iii) the expansion of the illegal drug trade in some areas within the pioneer front that, encouraged by the precarious actions of the state, rising unemployment, and the fragmentation of the socio-spatial urban fabric, lead to violence in the region [7].

Studies that treat the diffusion of homicides occasioned by economic processes in land occupation fronts are scarce. However, some researchers have investigated violence stemming from determinants exogenous to the conflict process and considered the relationship between violent homicides and socio-economic factors. Bursik and Grasmick [8] and Morenoff *et al.* [9] revealed that structural indicators—low socio-economic status, residential mobility, and a population’s ethnic characteristics—are significant determinants of crime or the victimization of individuals in space. Kawachi *et al.* [10] highlight social inequality as an important explanatory component of violence, regarding violence in the Amazon the same explanatory components are observed Hèbette [11], Costa [12], Martins [13], Mello [14], Hèbette [15], Loureiro [2], Castro [16] and Sant’Anna *et al.* [17] .Ye *et al.* [18] analyzed the relationship between homicide rates and socio-economic factors between 1960 and 1995 in urban space. The spread of homicides in Brazil is associated with migration, the rupture of family and social structures, the absence of state institutions, and impoverishment [19,20] are factors that can affect the local social dynamic and introduce violent acts into metropolitan and rural regions, such as in the expanding areas of the economic frontier.

However, unlike infectious diseases, the diffusion of homicides is different and peculiar. The spread of infectious diseases is not defined by a single individual; acts of violence can be committed by political groups and organizational interests [18]. In addition to the actions of individuals and social groups, the geographical context has a pertinent role in the diffusion of violence. The dissemination of homicides is defined by the economic structure, the mobility of the population, and the presence of existing public policies in the space. Socio-economic difficulties impede social organization and favor the expansion of homicidal violence. Low-income communities are often marked by a weak organizational base that lacks the financial and human capital resources to identify and protect their interests. These factors affect the population in different ways and intensities in accordance with the vulnerability of the social groups, which is associated with their inclusion in place and in society.

Homicide arises as a striking contradiction that characterizes and aggravates the inherent instability of pioneer front areas. Homicides occur parallel to the reproduction of the same economic and socially unjust structure that still organizes the Brazilian territory [21]. These are old conflicts, and the public is no longer surprised by the sequence of notorious events, such as the death of Chico Mendes in Acre (1988), the massacres of rural workers in Corumbiara, Rondônia (1995), and Eldorado dos Carajás, Pará (1996) and most recently, the assassination of Dorothy Stang in Pará (2005) [6]. The deaths arising from conflicts in the countryside and in the city throughout the country demonstrate a temporal and spatial continuity of the processes of violence, particularly in the metropolitan regions and expansion areas of the economic frontier [22].

This work investigates the diffusion of cases of homicidal violence in the Amazon in the last few years (1991–2010) based on population mobility, the expansion of agro-mineral activities in the region, and the routes of occupation. By explaining the diffusion and spatial patterns of homicidal violence, the study reveals the expansion of vulnerable areas, which can provide guidance for targeting preventive actions at the regional level.

## 2. Methodology

Homicide data were obtained from the Mortality Information System (Sistema de Informação sobre Mortalidade—SIM) managed by the Department of Information of the Unified Health System (Departamento de Informática do Sistema Único de Saúde—DATASUS, Brasília, Distrito Federal, Brasil). The database contains information obtained through the classification criteria of the basic cause of death present in the International Classification of Diseases, Ninth Revision (ICD-9) (E960 and E969; E985 and E986), for the most recent years and for the pre-1996 records that used the ICD-10 (X85 and Y09; Y22 and Y24, and Y28), with four different homicide categories—deaths resulting from legal interventions or military operations; deaths by homicide; injuries by firearms with unknown intention, and injuries by punctures-cuts with unknown intention. Homicide rates were calculated by the ratio between the numbers of deaths in the population per 100 thousand inhabitants.

To give greater stability to the indicators and minimize the effects of fluctuations, the homicide rates were calculated by means of the arithmetic mean of different periods (1991 to 1995, 1996 to 2000, 2001 to 2005, and 2006 to 2010) using as a denominator the estimated population in the central year of each period. The periods were organized in a geo-referenced database according to municipality of residence. An indicator of the temporality of the homicides was also created by calculating for each municipality the first year in which the number of homicides exceeded ten records. The inclusion of this indicator in the map allows for visualizing the dynamics of the spatial diffusion process [23].

To build the maps, the municipal grid of the Legal Amazon and the coordinates of the municipal capitals in 2010, which were provided by the Brazilian Institute of Geography and Statistics (Instituto Brasileiro de Geografia e Estatística-IBGE, Rio de Janeiro, Brazil) and mapped using the program MapInfo 9, were used. For the analysis of spatial patterns, the inverse squared distance weighted (IDW) interpolation method was employed [24] using the *Vertical Mapper* tool. In this type of interpolation, the intermediate values of the data are preserved and the final result is a continuous surface of smoothed indicators, minimizing the contrasts between the municipality polygons [25].

The application of the IDW method adopts a moving circle to identify each node of a grid created along the distribution of points, and weights are assigned to points located at a pre-defined distance. The interpolated values represent the weighted mean of values surrounding each grid cell. The weights attributed to the points were based on the distance from the cell. The IDW method is most commonly applied to data that can be highly variable over short distances [26]. The parameters required for the use of the IDW technique include the search radius, the grid display radius, and the cell size and the exponent of the interpolation equation. In the present study, parameters that covered the entire analyzed region were adopted while considering the mean distance between municipal capitals, equivalent to 216 km.

Maps with interpolated homicide rates were generated for the three analyzed periods (1991–1995, 2001–2005 and 2006–2010), allowing for the visual examination of changes in the spatial distribution of homicide rates on a regional scale.

This methodology was applied with the objective of seeking evidence of the spatial diffusion of homicides on the agricultural frontier, which encompasses risk areas related to migration and the recent occupation of economic activities in pioneer areas. However, these social and political processes are difficult to understand through secondary data. Nonetheless, the expansion of the economic front caused great social inequalities that largely support explanations of homicidal violence in Brazil. Additional social determinants have contributed to the increase in homicides, such as impunity and obsolescence, the devaluing and corruption of public safety institutions, the loss of ethical values, drug trafficking, debt collections, and corrupt organizations, such as those employing hired killers [27,20]. In the Legal Amazon, some of these socio-economic and environmental indicators were used to analyze the problem of violence as a socio-spatial phenomenon.

Environmental and socio-economic information was used as spatial data layers to aid in the interpretation of the data. This information was obtained from different sources, such as mining areas Geological Service of Brazil (Serviço Geológico do Brasil) [28], indigenous reserves (National Indian Foundation (Fundação Nacional do Índio—FUNAI, Brasília, Brazil) [29], the Ministry of the Environment (Ministério do Meio Ambiente—MMA, Brasília, Brazil), and Legal Amazon logging centers [30]. Each of these layers was obtained in the *shapefile* format. The following data were used for the land use analysis: production of plant extraction and silviculture; number of trees felled and the volume of wood in logs used [31] (Agricultural Census 1991 to 2010, Brazilian Institute of Geography and Statistics (Instituto Brasileiro de Geografia e Estatística-IBGE, Rio de Janeiro, Brazil); cattle herd (Agricultural Census 1991 to 2010, Brazilian Institute of Geography and Statistics (Instituto Brasileiro de Geografia e Estatística-IBGE, Rio de Janeiro, Brazil) [31], and soy production areas in the Legal Amazon (Agricultural Census 2006, Brazilian Institute of Geography and Statistics (Instituto Brasileiro de Geografia e Estatística-IBGE, Rio de Janeiro, Brazil) [32] . Using the absolute values of the variables (soy production per hectare, silviculture production per hectare, and number of cattle slaughtered per hectare), interpolations were calculated using the program MapInfo, generating different smoothed layers in MIF format.

One of the factors that contributed most to the economic and environmental changes in the region is the construction of a set of highways that, next to the Amazon River waterway, constitute axes of regional occupation. Therefore, layers corresponding to the primary highways of the region were obtained from the following highways: Brasília Distrito Federal to Belém-Pará (BR-153/010), constructed in 1960; the Trans-Amazonian Highway from Cabedelo-Paraíba to Lábrea-Amazonas (BR-230), which began construction in 1972; Cuiabá-Porto Velho-Rio Branco (BR-364), 1983; Porto Velho-Manaus-Boa Vista (BR-319/174), 1968/1976, and Cuiabá-Santarém (BR-163), 1972. This information was obtained from the National Department of Transportation Infrastructure (Departamento Nacional de Infraestrutura de Transporte—DNIT, Brasília, Brazil) [33]. The paths of these roads were used to plot longitudinal profiles of homicide rates with the aid of the *cross section* tool of the *vertical mapper* application in MapInfo.

The correlation between population mobility and murder has been observed in the study of Barcellos *et al.* [34] on the occurrence of homicides along the BR-163 path, between 1980 and 2005, which revealed the existence of an axis between the municipalities of Mato Grosso to the state of Pará, where he was constant the occurrence of homicide cases over the study period. Nevertheless, the region is a stage of a considerable pendulum moviment, the mobility of people in search of land and jobs, that is, in Pará stretch of the land market has not yet been stabilized and, still, there are areas available for expansion of economic activities related to the deployment lumber exploration, mining, livestock and soy, which, according to the authors would increase the distribution of homicide cases among the municipalities that are tangential to the highway, where the pioneer front is expanding. The homicide violence in the state of Mato Grosso, is on the mobility of people who are in search of land and work stations, since they were expelled from their territories due to deployment of public and private colonization projects without opportunities people moving from the countryside to the urban periphery of cities, an unemployed citizen living in a degraded environment in a state of poverty and misery, feels no identity, pressed and aggressive, this may lead you to commit crimes against other people, and even against his own life, which according to the authors would increase the occurrence of violence in cities located along the highways.

The agro minerals projects in the Amazon usually cross the forests and involve deforestation and displacement or relocation of residents, as well as the flow of migrant workers to new expansion areas and urban centers [21,35]. To support the development of economic corridors, road terrestrial networks were created in front pioneer, which catalyzed migration on a large scale to scale regions of implementation of agri-livestock projects. These areas pose a challenge not only for the implementation of economic activities and for migrant fixation, because existing socioeconomic difficulties in occupancy runners prevent the social organization of workers and encourage the spread of deadly violence. In occupancy axes, violence in rural areas is associated with the land market speculation and the confrontation by the acquisition of areas endowed with natural resources. On the outside the fences of the countryside, cities are home precariously temporary workers on the outskirts, in a degraded environment in a state of poverty and misery, which could expose the unemployed citizen to aggressive and violent behavior, in order to survive that it is denied in society. The displacement along the roads, in search of land and labor, can put the most vulnerable, *i.e.*, less conflictive areas in a high risk environment for conflict and divergences.

## 3. Results and Discussion

### 3.1.Spatial Diffusion of Homicidal Violence in the Amazonian Pioneer Front

**Figure 1:**
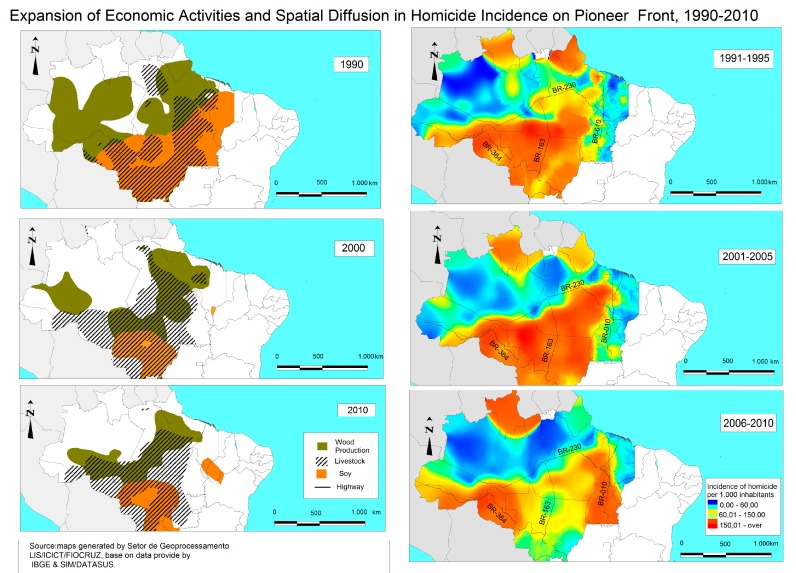
Maps of homicide rates in the Legal Amazon by period. * The average rate of homicides values corresponds: Low 0–60 (per 100 thousand inhabitants); Medium 60–150 (per 100 thousand inhabitants); High < 150 (per 100 thousand inhabitants).

Depending on the degree of development of the land occupation fronts, the interaction of the old occupation fronts with other recently occupied areas explained the inverse spatial pattern over time of the spatial distribution of homicides. The spatial patterns of death by homicide in the Amazon are shown in Figure 1.

Almost all of the states in the Legal Amazon exhibited an increase in the homicide rate in every period. The inverse spatial pattern of the spatial distribution of the cases over time was explained by the degree of development of the land occupation fronts and by the interaction of the old occupation fronts with recently occupied areas, with the exception of the municipalities in the state of Rondônia, which exhibited a decline beginning in the second period (2001–2005). The states of Mato Grosso, Rondônia, the south of Acre, the southeast of Pará, Roraima, and Amapá exhibited the greatest increase in incidence rates.

The expansion of homicides followed the areas of establishment of productive activities (such as wood, livestock, and soy extraction). The region containing the arc of deforestation recorded the greatest increase in homicide rates in the second period, and the areas with the greatest number of deaths by homicide in the entire period were the states of Mato Grosso, Rondônia, the south of Acre, and the south and southeast of Pará. At the time, the spatial diffusion of homicides continued to follow the expansion of the wood, livestock, and soy extraction activities. In the final period, after the intense and progressive pace of closure of the pioneering agricultural frontiers, the spatial spread of homicides in southern Mato Grosso exhibited an inverse pattern in space and time, and the states of Acre and Tocantins exhibited an expansion due to the extension of economic activities.

In the period of 1990–1995, the conty with the highest murder rate in Mato Grosso was Juína, in Rondônia the county of Machadinho D’Oeste, in South Acre the county of Rio Branco, in southeast Pará the county of Xinguara, in Roraima the county of Boa Vista and Amapá the county of Macapa. Between 1996–2000, the highest rates were presented in Mato Grosso was Cotriguaçu, followed by Rondônia the county of Cujubim, in Acre the county of Rio Branco in eastern Pará the county of Eldorado do Carajás, in Roraima the county of Mucajaí and Amapá the county of Calçoene. In last period, the county with the highest murder rate in Mato Grosso was Aripuanã, in Rondônia the county of Alto Paraíso, in Acre the county of Brasiléia in west of Pará the county of Altamira, in Roraima the county of Caroebe and Amapá the county of Laranjal do Jari.

**Figure 2:**
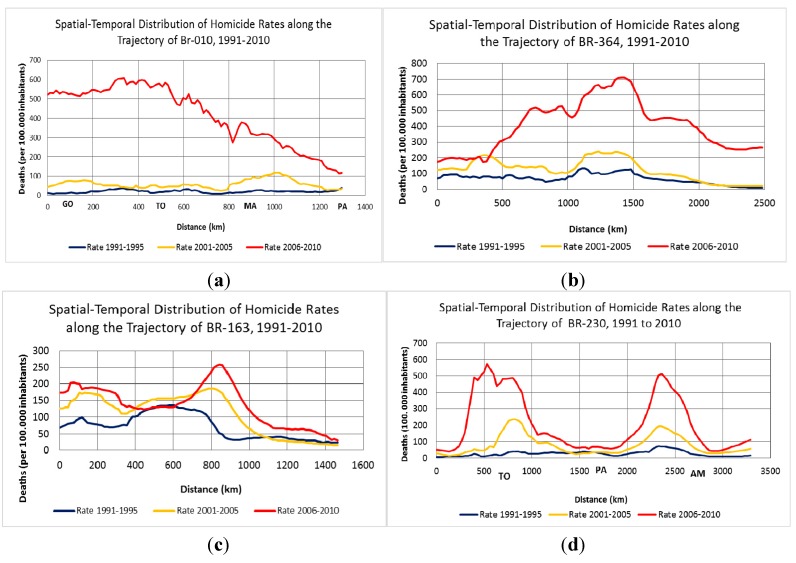
Interpolated homicide rates along the primary highways of the Legal Amazon (BR-010, BR-230, BR-163 and BR-364). Source: SIM/DATASUS (http://sim.saude.gov.br/default.asp).

Given the above, the spatial patterns of deaths from aggression in the municipality of the region can be seen in Figure 2. Note that, despite the color scale is the different shades, the occurrence of events intensity intervals vary between maps, watching -If greater range of homicides in the areas of influence of the roads.

From 1991 to 1995, the municipalities with the highest mortality rate by homicide in the state of Amapá were Laranjal do Jari, Oiapoque, Macapá, and Roraima. In the state of Roraima, the municipalities with the highest mortality right by homicide were Boa Vista and São Luiz. On the BR-010 route, the municipalities with the highest homicide rates in the state of Tocantins were Porto Nacional, Colinas do Tocantins, and Araguaína, as well as some other municipalities in the state of Pará, such as Paragominas, Xinguará, Conceição do Araguaia, Dom Eliseu, Belém, and Anandieua, and some other municipalities in western Maranhão state, such as Imperatriz, and Açailândia. In the municipalities situated along BR-163, the rapid increase in the homicide rates in municipalities in the state of Mato Grosso (such as Cuibá, Guaratã do Norte, Matapú, and Peixoto de Azevedo) and in the state of Pará (such as, Itaituba, Altamira, and Santarém) should be highlighted. The BR-230 highway experienced an increase in the homicide rate in the municipalities of Marabá, São Felix do Xingu, and Altamira in Pará state and Presidente Dutra, Barra do Corda, Grajaú, Arame, Açailândia, and Imperatriz in Maranhão state. The total number of deaths by homicide along a specific stretch of BR-364 was the largest in the period, particularly in the municipalities of Cuiabá, Comodoro, Vilhena, and Juína in Mato Grosso state, and Pimenta Bueno, Espigão D’Oeste, Ji-Paraná, Machadinho D’Oeste, Porto Velho, and Nova Mamoré in Rondônia.

The highest mortality rate by homicide between 2001 and 2005 was reported along the BR-364 highway (with more elevated rates in the municipalities of Cuiabá, Arenápolis, Tangará da Serra, Comodoro, and Juína in Mato Grosso and Pimenta Bueno, Cacoal, Ji-Paraná, Machadinho D’Oeste, and Porto Velho in Rondônia), followed by the BR-230 highway running through the states of Maranhão, Pará, and Amazonas (with higher homicide mortality rates in the municipalities of Tuntum, Barra do Corda, Açailândia, Imperatriz, and Itinga do Maranhão in Maranhão and Marabá, Itupiranga, Novo Repartimento, Pacajá, Jacundá, Nova Ipixuna, São Felix do Xingu, Altamira, and Medicilândia in Pará). A high homicide mortality rate was also found along BR-163 but only on the stretch that approaches Mato Grosso state (with high rates in the municipalities of Cuibá, Guaratã do Norte, Matapú, Peixoto de Azevedo, Rosário Oeste, Nobres, Nova Mutum, Diamantino, Tapurah, Sorriso, Sinop, and Itaúba). Although the BR-010 highway has the smallest number of municipalities with high homicide mortality rates, the Western Amazonian highway experienced the greatest increase in the homicide mortality rate in the entire period. The municipalities with the highest homicide mortality rates were Araguatins, São Bento do Tocantins, Xambioá, Araguanã, Araguainá in Tocantins, Tucuruí, Rondon do Pará, Jacundá, Itupiranga, São Domingos do Araguaia, Eldorado dos Carajás, Conceição do Araguaia, Dom Eliseu, and Ulianópolis in Pará and Imperatriz, Açailândia, and Itinga do Maranhão in Maranhão.

There was a increase homicide mortality in recent years only on two highways in the region and a decrease in the states of Mato Grosso, Pará, and Maranhão. Thus, the mortality rate by homicide increased along the BR-010 stretch that passes through the state of Tocantins but not the states of Pará and Maranhão (which showed a decline in the homicide mortality rate). The increase in Tocantins was more pronounced in the municipalities of Rio da Conceição, Brejinho de Nazaré, Miranorte, Divinópolis do Tocantins, Aparecida do Norte, Itaporoã do Tocantins, AraguaínaSanta Fé do Araguaia, and Wanderlândia. Meanwhile, along BR-364, municipalities in the states of Rondônia and Acre demonstrated an important increase in mortality by homicide throughout the entire period, particularly Porto Velho, Candeias do Jamari, Cujubim, Alto Paraíso, Machadinho D’Oeste, Ariquemes, Ji-Paraná, Buritis, Nova Mamoré, and São Francisco do Guaporé in Rondônia, and Plácido de Castro, and Porto Acre and Rio Branco in Acre. Therefore, a decline in mortality by homicide was observed for the region as a whole, although an increase occurred in certain areas (Figure 1).

To illustrate the spatial pattern of the distribution of the homicide incidence rate, cross sections were created along the trajectory of the most important highways of the region (BR-010, BR-230, BR-163, and BR-364) that link the capitals to smaller cities (Figure 2). The “z” value was obtained by interpolating the homicide rates through the arithmetic mean of each period along these axes (the total distance for each of the highways) for 1991–1995, 2001–2005, and 2005–2010. The roads were used to explore the structure and spatial dependence of homicide incidence rates in the Legal Amazon.

Along each of the highways, the incidence rates were observed over all years. In all periods, the incidence rates were recorded along the trajectory of each highway. The first wave (1991–1995) of violence reached the cities near Porto Velho, Rondônia along BR-364 of Cuiabá, Alta Floresta, Sinop, Sorris, and Ipiranga do Norte in Mato Grosso and the cities along BR-163 of Itaituba, Belterra, and Santarém in Pará. However, the intensity of the violence remained low along BR-010 and BR-230. In 2001–2005, the waves of violence were more intense along BR-364 and BR-163, when the movement of the waves was observed in the capital and its outskirts, respectively, along BR-364 (Cuibá, Arenápolis, Tangará da Serra, Comodoro and Juína in Mato Grosso, Pimenta Bueno, Cacoal, Ji-Paraná, Machadinho D’Oeste, Candeias do Jamari, and Porto Velho in Rondônia) and along BR-163 (Cuibá, Guaratã do Norte, Peixoto de Azevedo, Rosário Oeste, Nobres, Nova Mutum, Diamantino, Lucas do Rio Verde, Sorriso, Sinop, and Itaúba in Mato Grosso). However, in the final period, there was a decrease in the intensity of the wave in the state of Mato Grosso. The waves moved toward urban areas, amplifying in intensity along all trajectories.

**Figure 3:**
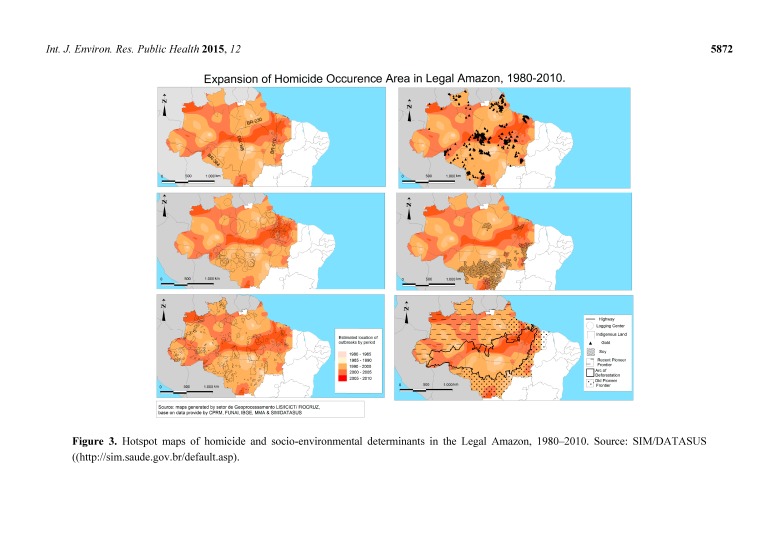
Hotspot maps of homicide and socio-environmental determinants in the Legal Amazon, 1980–2010. Source: SIM/DATASUS ((http://sim.saude.gov.br/default.asp).

Figure 3 shows the spatial diffusion of homicidal violence in the Legal Amazon. The map color gradation is the time variation of the occurrence of cases of murder. The lighter colors refer to the earlier years and dark colors are related to recent years. According to the map, the homicide outbreaks expanded their area of occurrence through phases of occupation of the pioneer fronts, signaling the expansion of outbreaks along the consolidated pioneer area and the area of the arc of deforestation. Some of the diffusion axes depicted in the figure correspond to major roads linking major cities to areas of logging and mineral exploration. The widespread diffusion occurred before 2000 and between 2000 and 2010. The concentration of outbreaks corresponded to an increase in violence in the areas where specific economic projects were established and in the region’s medium and large cities.

The outbreaks observed in 1980–1985 were concentrated in the northeast of Mato Grosso, in the south and northwest of Pará, in the south of Amazonas and in Acre, the pioneer area of the region at the time. Between 1985 and 1990, the outbreaks moved to the north of Pará (Itaituba and Santarém), northern Mato Grosso, and Rondônia, municipalities located in the exploration areas of gold and timber. In recent years, the most vulnerable areas were clustered in the deforestation arc in northern Rondônia, Roraima, Amapá, and northern Amazonas. Some of these areas currently correspond to the expanding pioneer area.

## 4. Discussion

The study of the spatial diffusion of homicidal violence in the Legal Amazon demonstrated that the propagation of waves of violence followed the sequence of occupation of pioneer areas, but there was an expansion to urban areas after the exhaustion of the pioneer fronts in the countryside.

The beginning of the 1990s witnessed the spread of deadly violence along BR-010, toward the southeast of Pará and west of Maranhão, reaching the oldest and most densely populated areas, such as the region of Bico do Papagaio and some medium-sized cities. Due to the selective migratory fluctuations of northeasterners and the implementation of extensive cattle ranching and accelerated deforestation, the occupation was marked by the growth of conflicts due to the struggle for land, rural murders, and instances of slave-like labor conditions that occurred in the region [2,3].

From 1991 to 1995, there was a marked increase in the incidence of homicides along BR-163, specifically in the north of Mato Grosso and in the southwest of Pará, which surpassed the rates along BR-364, with the state of Mato Grosso being the area with greater homicide incidence rates until 2005. The BR-163 route experienced an increase that can be explained, in the state of Mato Grosso, by process deforestation and conflicts caused by the dispute and possession of land. Meanwhile, in southwestern Pará’-Itaituba and Santarém, homicides occurred due to gold extraction in the mining areas [35]. Similarly, in the municipalities crossed by BR-364, the expansion of homicidal violence resulted from the existence of gold alluvial deposits in the Upper Madeira River.

With regard to the state of Mato Grosso, it was observed that during the construction of highways BR-163 and BR-364, private colonization projects were executed by public and private firms [36]. Some studies note that this factor stimulated changes in the reduction, expansion, and maintenance of agricultural frontier areas, leading to the enclosure of fields and redirecting the distribution of population flow to other regions of the Amazon, a result that contributed to the shift of violence from the areas of conflict and disputes over land ownership in rural pioneer areas to specific areas in medium and large cities in the state. There was a trend of escalation of conflicts involving migrants, which was accentuated due to the reduced potential for demographic absorption of most of the conflicts because of the expansion of urban social segregation and the risk of exposure to violence [37].

Thus, the dubious and contradictory character of government actions, which encouraged the expansion of agribusiness selectively protecting areas in the region, is evident. These political actions brought together and incited social groups with conflicting interests that resulted in the repetition of criminal practices characteristic of regional occupation and marked by the often-illegal appropriation of land for the purposes of land speculation and, strategically, for obtaining credit or for illegal logging [38,39].

Some studies have pointed to spatial changes in the shift in homicide rates. After 2005, new areas of environmental protection were created to manage the use of public forests and “strengthen civil society and local social movements” along the region’s highways [40]. There was a trend toward the stabilization of incidence rates in these areas, but the downturn in deadly violence was not homogeneous. In fact, homicides expanded to other areas because protected areas prevented the establishment of other economic activities there [41]. These conservation areas, despite playing an important role in forest maintenance, indirectly stimulated migration and the intensification of competition for natural resources and land in other locations of the pioneer area.

The results point to an increase in the incidence of homicides in the areas in which the state provided for the expansion of agribusiness through the implementation of programs such as the Plan for the Development of the Center-West (Plano de Desenvolvimento do Centro-Oeste, 2007–2020) and the Growth Acceleration Program (Programa de Aceleração do Crescimento –PAC, 2007–2010). Rondônia, Mato Grosso, and stretches of BR-163 showed an increase in homicides in the areas in which government intervention for infrastructure development is occurring. Although there was a decline in homicide rates in private colonization areas, new homicides increased in the municipalities for which developmental actions in the agribusiness chain were provided to improve the road network and increase production flow. Thus, paving the road to lower the cost of production encouraged migration and competition for land and natural resources along the highway.

Studies show a constant increase in the incidence of homicides along BR-364 and BR-230 since 1980. The increase in homicides resulted from the demarcation of lands on the borders of these highways that were shared according to social categories (large, medium, and small producers). At the time, the distinction between the type and nature of the lots supplied to settlers and business owners meant that small farmers had great difficulty in settling on the land, leading to the abandonment of the land and the search for new places to develop small-scale production. For this reason, the region was occupied by extensive cattle ranching, which appropriated larger properties and better infrastructure (with roads and electricity). This inequality in the distribution and quality of the lots intensified and even today explains the current conflicts over land ownership and the vulnerability in the occurrence of homicides on and around the two highways [15].

The Amazonian population occupation occurred at a faster rate than the country as a whole. It was marked by a high degree of urbanization. Just check out that in 1970 the population of the Amazon was composed of 63% of rural population and only 37% urban. In 1991 this ratio is reversed, and the population now lives in cities predominantly (56%) and, already in 2000, 69% live in areas defined as urban and 31% in rural areas [42,43,44]. Thus, the population growth in the region concentrated along the urban centers, with this rapid urbanization has caused problems of urban growth, disorganized, with slums, violence and lack of services and facilities to serve the population.

In recent periods migration is directed to specific projects linked to Avança Brazil, Brazil in Action and the Growth Acceleration Program, this project stimulate intraregional migration and reduce interregional migration, since then adopt another policy territorial occupation that interferes with the logic of populacional flows. In the period 2000–2010, data from the population census showed that interstate migration in the country has changed. In this time interval, 10.2 million people changed their residence state, and close to 1.6 million had as destiny the Brazilian Amazon, and of these, 795,000 were Federative Units exchanges in the Amazon itself. [45].

According to data from the population census, in the 2000s the Amazon Federation Unit (FU) the ones that received most migrants were Pará (392.000), Mato Grosso (343.000) and Maranhão (230.000). However, the states of Mato Grosso, Maranhão, Tocantins and Rondônia received many migrants from other Fus outside the Amazon. In the 2010 census, Pará and Amazonas were the states that received intra-regional migrants. Despite the Pará stand out as the main FU receiver of migrants, interregional terms, Pará was already losing population to FUs in the Amazon, especially Maranhão, Amapá and Amazonas [45]. Still, Pará is considered one of the states with the highest rates of violence in the region in the last three decades, jointly with Mato Grosso, Rondônia, Roraima and recently Tocantins.

It can be said that the actions filed by the state, roughly, in both peroxides contributed to the intensification of land conflicts, to promote, above all, business groups and large landowners. Thus, the conflict has been interpreted by the state technocracy as factors inherent in the modernization of agriculture, where land concentration would be the natural way of the land ownership at the border. So it can be concluded that violence in rural and urban areas in the Amazon are the result of a common matrix, which is the exclusion of access of land and social imbalances caused by it [45].

Thus, the process of inserting the Amazon in national and global structure was consolidated unevenly in spatial terms, enhancing the spatial differentiation and social exclusion, creating new forms of appropriation of natural resources - or repurposing the old—for new features and at the same time, marginalizing other.

The present study also revealed the occurrence of other patterns of conflict and violence that occur in the mining regions and in the cities connected to the drug-trafficking route. In these areas, the incidence of homicides is likely related to drug trafficking, illegal gun running, crime, and even violent means for solving personal disagreements [46].

The disparities in homicide incidence in the Legal Amazon can be understood in light of the dynamics of the extractive exploitation of timber. At the beginning of the extractive activity, loggers purchase exploitation rights from settlers in return for access to the land or improvements located along the highways and back roads. This exchange relationship can directly affect workers’ exposure to risk. When small producers do not meet the agreements made with loggers, conflicts and violence of every type can occur in the intermediary process of wood extraction in the region [47,17].

Additionally, many municipalities with high homicide rates are located in Indigenous Lands (IL) near the primary federal highways. The establishment of ILs can lead to various changes, such as hampering access to land and natural resources, causing exhaustion of pioneer areas, and disputes in certain areas. An example of these changes can be seen in the conflict that occurred in the ILs of the Cinta Larga tribe, located near BR-364. The first conflicts in this area involved the rubber tappers and, later, the illegal extraction of diamonds by miners. In 2004, the dispute over the clandestine extraction of diamonds resulted in the massacre of 29 miners. The deaths of the miners provoked a strong reaction from the local population against the Cinta Larga tribe, and some tribe members were killed in revenge [48].

The incidence of homicide was high, particularly in the eastern portion of highway BR-230, in the state of Pará, between the cities of Marabá, Altamira, and Itaituba, places in which the transformation of the social and economic structure have been more dynamic, in contrast to the state of Amazonas, where long stretches of the highway showed little change in the homicide rate. That area has socio-economic characteristics that reduce its vulnerability compared to the remainder of the region. Access difficulties and low density of economic activities have relatively preserved the western portion of the highway [49,50]. In this case, the isolation population groups and low economic attractiveness can, ironically, act as a protective factor for local populations against homicidal violence [34].

Figure 3 indicates that the first wave of diffusion of violent outbreaks is related to land disputes, timber exploration, mining and, afterward, soy. The social disorganization and reorganization caused by these activities, associated with the absence of the state, are factors that explain, in part, the occurrence of deaths by homicide. Additionally, the second phase of the spread of the outbreaks occurred in the more urbanized regions of the frontier, where in recent year’s homicide outbreaks appeared and were concentrated in the cities, thus depicting the transfer of violence from the rural areas to the urban areas of the Legal Amazon.

This process occurs when, for example, a heterogeneous population migrates to cities and settles in slums and temporary camps outside city fences. The decline of extraction cycles, the decadence of the boom towns [51], and the idleness of the agricultural sector bring to large and medium cities large numbers of new inhabitants who are vulnerable to violence [52].

The homicidal violence in the regional urban space can occur when the spatial dispersion of drug trafficking operations promotes a shift of violence to cities and the localities tied to the drug route and narcotics economy [53].

The permanent areas of violence (Figure 3) coincide with the circuits of timber extraction, primarily in Altamira and São Félix do Xingu, where the “Kings of Mahogany” and of other noble wood species operates within the indigenous reserves. The other fronts of expansion of legal or illegal timber businesses occur in Terra do Meio- Pará, Novo Progresso- Pará, and Rondônia [54].

According to the mapping of the municipalities that used interactive operations between different layers with the layers of homicide occurrence, areas of recent expansion of violence are found, in general, within the so-called arc of deforestation, in mining areas, and near the timber exploration centers. The areas of homicide concentration coincide with the occurrence of slave labor in the north of Amapá, Maranhão, Mato Grosso, Pará, Rondônia, and Roraima and occur in the extractive mining regions in indigenous areas, such as southern Pará, which may involve any number of conflicts and the absence of citizenship rights [2,3].

However, the areas of soy cultivation exhibited a reduction in homicides in recent years in the state of Mato Grosso, following land revaluation [39,55]. There was a lower concentration of homicides in areas where new municipalities were established between 1997 and 2001, with a strong concentration in the north of Mato Grosso, Rondônia, Roraima, the southeast of Pará, and the south of Maranhão, which indicates the intensification of political movements and the autonomy of the districts in relation to the distant capitals of the pioneer municipalities.

The creation of municipalities was partly fueled by rising incomes, populations, and the formation of city networks that came with the creation of new urban centers connected by roads. In this sense, the consolidation of local economic activities can help reduce violence by settling migrants and establishing institutions that overcome the historic lack of state actions in the region. The explosive character of these changes, with rapid growth followed by the decline of economic activity, has hindered sustainable development and aggravated the vulnerability of the region’s traditional populations [46,51].

The most recent data indicated a constant decrease in the incidence of homicides; the reduction in the homicide rate in Amapá, Amazonas, Mato Grosso, and southwestern Pará was accompanied by an increase in other areas. However, Roraima, Southeast Pará, and Southern Acre demonstrated an increase in the homicide rate between 2006 and 2010, although the increase was less pronounced for Rondônia compared to other years.

It should be noted that one indicator alone cannot explain a social problem as complex as homicidal violence; confounding variables must be considered. For example, studies indicate that initially, soy production zones and zones where timber is illegally extracted are prone to violence that can trigger the occurrence of homicides [6,57]. However, after the initial phase of implementation of these economic activities and, consequently, the regularization of the land market, the incidence of homicides in these areas stabilizes.

The studies examining the correlation between homicide risk and independent variables should be oriented toward a combination of factors that are necessary to understand the increase of risk of conflict and violence in pioneer areas. Social, economic, and environmental determinants operate by means of processes closer in order and distance, following a sequence until reaching their biological outcome: death by homicidal violence. For deaths by aggression to occur, a series of environmental processes and factors that affect the frequency and distribution of homicides are necessary, such as social inequality, population structure, population density, economic occupation, use and ownership of land, deforestation and unemployment. A change in any one of these environmental factors can affect the incidence of homicidal violence.

**Figure 4:**
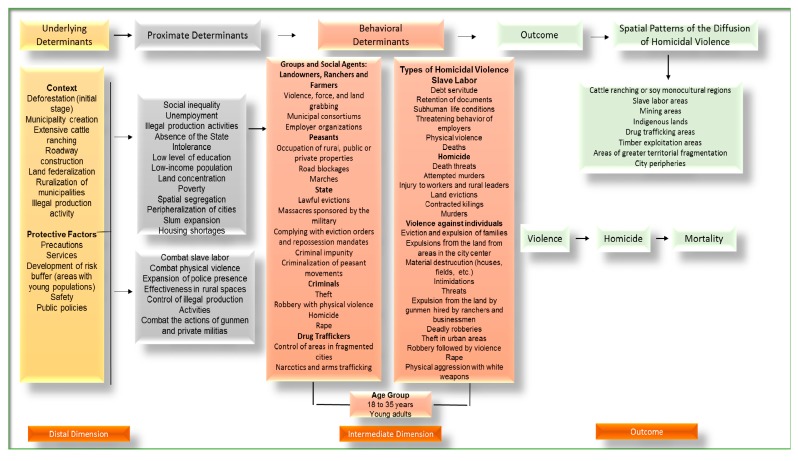
Proximate-determinants conceptual framework for factors affecting the risk of violence in the regional space.

Figure 4 shows the levels of proximate determinants in the framework of the determinants of violence in the regional space, which link the social and economic system, at left, with the ecological system, at right. The quantification of the effects of proximate determinants on vulnerable populations and their outcomes (*i.e.*, mortality) provide important information for the implementation of policies for health promotion and the prevention of homicidal violence.

In proposing this structure of determinants of violence, the study identified interactive processes that can aggravate or mitigate the manifestation of violence on a regional scale.

## 5. Conclusions

The distribution of homicides closely follows the penetration axes and economic expansion fronts in the region. However, the causes of violence are related to specific contexts in each area. The peculiarities and characteristics of each portion of the space should be studied to understand the possible socio-environmental determinants of each of these events.

The opening of the large highways, such as Belém-Brasília, the Trans-Amazonian Highway, Cuiabá-Porto Velho, and Cuiabá-Santarém allowed family farms, large-scale farmers, and businesses in search of land to penetrate remote areas, exposing forested areas to the action of these actors. The regional space was divided into large tracts and small plots along the highways, and the landscape underwent rapid and intense modifications. Consequently, the areas along the great penetrating axes (highways) became the settings of disputes over land ownership between indigenous people, traditional populations, small farmers, migrants, business owners, and the state [58,59].

The intermediate pioneer area (deforestation arc), which represents the first stages of economic expansion, altered values and promoted violence in the countryside because the occupation processes were affected by the definition of property rights, the structural characteristics of land concentration, and the set of public policies for the Amazon [6].

The analysis of the spatial distribution of deaths by homicide is consistent with the literature on the political, social, and economic determinants of the region’s expansion fronts [22,15,60,]. Since the 1970s, there has been a shift of the main locations of violence from rural areas in the Northeast to the Amazon. Deaths across Brazil due to conflicts in the field indicate a temporal and spatial continuity of the aforementioned processes. In the Amazon, the critical zone extends to southeast Pará, northern Mato Grosso, eastern Roraima, and central Rondônia.

The study identified that the highest values of average homicide rate in the Amazon are in the arc of deforestation. This was initially occupied for the logging. Then the pasture with the implementation of livestock and finally, in areas with affinity and greater economic dynamism, were introduced mechanized cultivation of soybeans, corn, cotton and rice. In this uninterrupted cycle of land appropriation, control and ownership of the economic frontier were marked by grabbing of public lands and their private and illegal appropriation generating violence on the border. Outside this area are located in the municipalities of Amapá, by the occurrence of exploitation of mining, and and also the municipalities of Roraima, which occurs the introduction of rice cultivation and extraction of precious stones (diamond) and gold.

The study pointed out different levels of distribution of violence with a very unique cases density, which includes municipalities in the three areas of occupation pioneer Amazon front: the arc of deforestation consolidated populated areas and the expansion of settlement sites. In each of these parts of the economic front, over the years, it was noticed the increase in rates in the three areas, with greater intensity in the arc of deforestation, as the most dynamic area of the border and where the main economic enterprises are situated. In this area, currently, the violence is concentrated in medium and large cities, but previously was concentrated in rural areas. This confirms the pattern of spatial distribution of homicide cases, marked initially by their spread in the areas of expansion of the population, predominantly rural violence and then shifts to a stage of stability, in which the dispersion of cases moves the countryside, through population dynamics, that by failing to settle in the territory, where the labor market and land became scarce, moves to areas of medium-sized cities and urban neighborhoods of large cities, probably contributing to expansion conflicts related to personal disagreements or crime.

Starting in the 1970s, new cities emerged, mostly within the spheres of influence of the three major highways built by the federal government after 1960, in the areas of dry land between the main rivers of the Amazon region: the Belém-Brasília, the Trans-Amazonian, and the Cuiabá-Porto Velho highways. The occupation of areas along the highways established precedents for a speculative process, often illegal and violent, of land delimitation and occupation. A legacy of land disputes, fraudulent titles, and rural violence still plagues these areas [49]. Some of the main determinants of the spatial diffusion of homicides in the region can be summarized, allowing the geographical dimension of violence to be understood [60]:

(a) The growing land dispute triggered by companies and land grabbers who formed private militias. Private “surveillance” or “security” appeared in the region to protect large tracts of land acquired by large-scale farmers. However, this land is kept idle or unproductive and is protected against squatters [2].
(b) The restriction of the freedom of rural migrant workers in search of land and jobs, who cannot pay off their unmanageable debts to large-scale farmers. This situation has kept these workers under the control of armed employees who use force, intimidation, and murder to control enslaved laborers [3].
(c) The presence of illegal production activities, such as logging, charcoal production, and gold exploration can result in the formation of illegal labor relationships without formal work contracts, which leads many workers to become victims of violence and slavery [3].
(d) The fragmentation or amalgamation of municipalities promoted the reformulation of the territorial fabric. The creation of new municipalities resulted not only in the economic growth of the regions on the pioneer front, but in the political movement of some of the inhabitants. The pioneering spirit, the independence of will, the courage to confront difficult situations took part in the same social, political, and economic arena, whose dark side is the permanent presence of political crime in the region [3].
(e) The expansion of the coca and cocaine economy that reached cities and some areas of the Amazon, such as protected areas and indigenous lands, which were integrated into the trafficking route. The state’s repressive actions, promoted through attacks on laboratory areas where narcotics are sold and circulated, as well as the activities of traffickers who in the distribution scheme enter into conflict for the definitive control of certain areas to sell drugs [53].
(f) The processes of social segregation in urban spaces generated by the formation of cities in public or private colonization projects. In the cities of the colonization projects, the urban space is divided and controlled by business people from the planning sector who, when planning the occupation of empty spaces, use violence to reserve the central areas of the cities for companies [37].
(g) Agrarian conflicts in the areas that received many fiscal incentives from the federal government. In these areas, a large portion of homicides occur in areas of allocation and distribution of federally funded resources, where activities linked to agricultural projects and forest management occurred [12].
(h) The penetration of important migratory fronts in the region. In the Amazon, migration to the frontier is a continuous process that, even today, advances into the Amazon’s interior, leading old and new migrants in search of land. In such cases, the migrant’s establishment is hindered by various obstacles that lead to a multifaceted (spatial, professional, and social) migrant mobility: (1) the competition between the strong and the weak for land ownership, which results in bloody conflicts that are not limited to the first occupation; (2) the ecological constraints and lack of financial and technical resources necessary to adjust local environmental and economic conditions, and (3) the harshness of life in the countryside, isolation, hard work, disease, and poverty. From those conditions stem the migrant’s aforementioned multifaceted mobility [62].
(i) The increase in deforestation, which results in an increase in conflict. Through deforestation, it is possible to obtain property rights with the expectation of sale later to other economic agents. As property rights are defined, the inhabitants that previously occupied the land are expelled through the use of violence [6].

This study suggests that high homicide rates in the Amazon are correlated specifically with the opening of the roads where the main factors responsible for expansion occurred with land speculation, competition for natural resources, urban bloat, and the absence of the state in economic frontier areas.

According to the research, the highest homicide rates are located in the counties cut the roads, specifically, those cut by paved federal and state roads. This was due to groups with interest in mining, mineral and forestry, livestock or migrates to the region targeted by the opening of roads built to the new areas access route, arriving at these sites the conflict and the dispute over the ownership of land in the expansion fronts. Recently, this occupation logic remains, and the highways are used to move the labor market and land in the region, a fact explained by the depletion of old areas with soil degradation and the valuation of the land, which stimulate the migration of the population to neighboring areas to the extent that these counties are incorporated into the dynamics of more consolidated frontier by the intensification of land use for purposes mainly agricultural. This is the great advantage of these areas in relation to others, whose municipalities have little accessibility are directly dependent on river transport, being less affected by the expansion of economic activities and conflicts associated with them.
